# [Retracted] SIRT4 overexpression protects against diabetic nephropathy by inhibiting podocyte apoptosis

**DOI:** 10.3892/etm.2024.12779

**Published:** 2024-12-09

**Authors:** Jian-Xia Shi, Qi-Jin Wang, Hui Li, Qin Huang

Exp Ther Med 13:342–348, 2017; DOI: 10.3892/etm.2016.3938

Following the publication of the above paper, a concerned reader drew the Editor’s attention to the fact that the right-hand pair of flow cytometric plots shown in Fig. 2E on p. 345 [showing the results of the ‘Glu (30 mM)+Vector’ and ‘Glu (30 mM)+SIRT4’ experiments] contained unexpected areas of similarity / identity; even though the gate settings were different; in addition, certain of the western blot data were strikingly similar to data that appeared in articles relatively soon afterwards submitted to other journals written by different authors from different research institutes, ultimately calling into question the source of the data concerned.

Following an internal enquiry, the Editor of *Experimental and Therapeutic Medicine* was able to verify the claims made by the concerned reader; therefore, in view of the abovementioned anomalies that were identified in Fig. 2E and owing to an overall lack of confidence in the presented data, the Editor has decided that the paper should be retracted from the Journal. The authors were asked for an explanation to account for these concerns, but the Editorial Office did not receive a reply. The Editor apologizes to the readership of the Journal for any inconvenience caused.

